# Return of Norovirus and Rotavirus Activity in Winter 2020‒21 in City with Strict COVID-19 Control Strategy, China

**DOI:** 10.3201/eid2803.212117

**Published:** 2022-03

**Authors:** Martin Chi-Wai Chan

**Affiliations:** Independent research scientist, Hong Kong, China

**Keywords:** COVID-19, coronavirus disease, severe acute respiratory syndrome coronavirus 2, norovirus, rotavirus, viruses, respiratory infections, seasonality, viral gastroenteritis, zoonoses

## Abstract

A rapid decrease in viral gastroenteritis during winter 2019–20 and a return of norovirus and rotavirus activity during winter 2020–21 were observed while multiple nonpharmaceutical interventions for coronavirus disease were in effect in Hong Kong. The initial collateral benefit of coronavirus disease countermeasures that reduced the viral gastroenteritis burden is not sustainable.

The unfolding novel coronavirus disease (COVID-19) pandemic is an unprecedented public health crisis in the modern history of humankind. One collateral consequence of this pandemic is the concomitant rapid decrease in the incidence of viral gastroenteritis in the first year of the pandemic, as observed in many countries, such as China (*1*), the United States (*2*), England (*3*), Germany (*4*), Japan (*5*), and Australia (*6*). The most likely explanations were reduced testing capacity that led to underreporting and wide implementation of nonspecific nonpharmaceutical interventions for COVID-19, such as frequent handwashing and physical distancing, that reduced human-to-human transmission of different viruses.

Hong Kong is a metropolitan city in southern China and has been continuously implementing stringent and effective elimination (also known as zero COVID-19) strategy to suppress importation and local spread of COVID-19 since the start of the pandemic. Local routine laboratory syndromic surveillance for viral gastroenteritis remained largely unaffected during the pandemic, and testing capacity for common diarrheagenic viruses was only mildly reduced, providing a well-controlled setting to study the epidemiology of viral gastroenteritis in the COVID-19 era. This report compares the activity of norovirus and rotavirus in winters 2019–20 and 2020–21 in Hong Kong while stringent social distancing and continual zero COVID-19 control strategy were in effect in the city.

## The Study 

Local territorywide monthly laboratory data on PCR-based detection of norovirus and rotavirus, the 2 leading causes of viral gastroenteritis (*7*), are publicly available since January 2013 from the Centre for Health Protection of Hong Kong (equivalent in function to other national public health agencies, such as the China Centers for Disease Control and Prevention) (*8*). Laboratory data for less common diarrheagenic viruses, including sapovirus, astrovirus, and enteric adenovirus, were available from May 2017 onward. During January 2013‒September 2021, a total of 104,187 stool specimens collected from sporadic and outbreak case-patients who had acute gastroenteritis were tested (Figure, panel B). The median number of specimens tested each month was 1,008 (interquartile range [IQR] 912‒1,114) before the COVID-19 pandemic and 872 (IQR 784‒990) during the COVID-19 pandemic. Although an average of 13.5% fewer stool specimens were tested during the pandemic (p<0.01 by Mann-Whitney U test), the reduced sample sizes were still of sufficient power to detect >1 positive specimen under a virus prevalence as low as 0.5% (namely 1 in 200) at a 95% confidence level.

**Figure:**
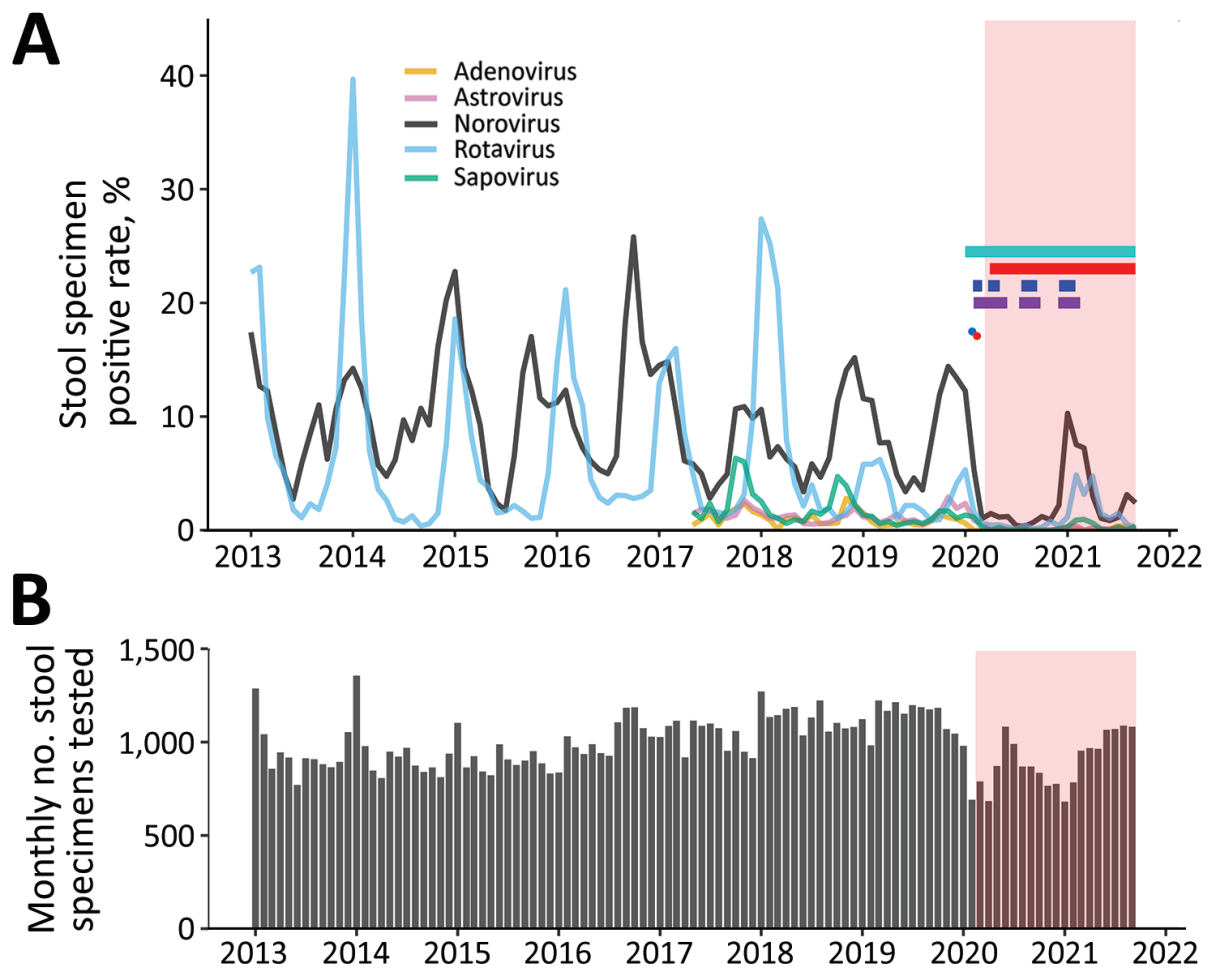
Positive rates for 5 common viral causes of acute gastroenteritis in stool specimens and total number of specimens tested from routine, territorywide, PCR-based laboratory syndromic surveillance data, Hong Kong, China, January 2013‒September 2021. A) Monthly positive rates. Data for sapovirus, astrovirus, and enteric adenovirus were available for May 2017 onwards. The first imported COVID-19 case (blue dot) was reported on January 23, 2020, and the first locally acquired case (red dot) was reported on February 4, 2020. COVID-19 was declared pandemic by the World Health Organization on March 11, 2020. Colored horizontal bars indicate the periods of major nonpharmaceutical interventions for COVID-19 in the city, including universal mask-wearing outside homes (aqua), prohibition on group gatherings of >4 persons in public places (red), work-from-home arrangement for civil servants (blue), and school dismissal (purple). Pink indicates time of the COVID-19 pandemic. B) Monthly number of stool specimens tested. COVID-19, coronavirus disease.

Monthly positive rates of the 5 common viral causes of acute gastroenteritis are provided (Figure, panel A). During winter 2019–20, the positive rate of rotavirus decreased abruptly from the peak by 70% during February 2020, shortly after the initial global spread of COVID-19, and remained at a much lower level of 0.1%‒0.6% through September 2020, compared with a median of 5.4% (IQR 2.8%‒13.4%) during the same period in the previous 7 years. The winter 2019–20 rotavirus season ended ≈2 months earlier than usual.

The observed lower positive rate might be confounded by the decreasing trend in recent years with the availability of 2 rotavirus vaccines: RotaTeq (Merck and Co. Inc., https://www.merck.com) and Rotarix (GlaxoSmithKline, https://www.gsk.com), both licensed for use since 2006 in private clinics, but not yet included in the local childhood immunization program. Therefore, I examined data further for cases of norovirus, for which no effective antiviral drugs or vaccines are available. Likewise, norovirus positive rates decreased sharply from the peak by 56% during February 2020 and remained at a much lower level of 0.3%‒1.5% through September 2020, compared with a median of 6.4% (IQR 5.2%‒10.0%) during the same period in the previous 7 years. The winter 2019–20 season for norovirus ended almost 3 months earlier than usual. 

I also reviewed data for sapovirus, astrovirus, and enteric adenovirus. These viruses became hardly detectable at the start of the COVID-19 pandemic, showing a positive rate of persistently <1% throughout 2020 and 2021.

In winter 2020–21, a typical seasonal peak of norovirus that had a positive rate of 10.3% was observed in January 2021, a rate comparable with the median of 14.4% during the previous 7 winter seasons. Likewise, a typical seasonal peak of rotavirus with a positive rate of 4.8% was observed in February 2021, highly comparable with the rates of 5.3% and 6.2% in the previous 2 winter seasons, albeit on a progressively decreasing trend in recent years. These data indicated active circulation of norovirus and rotavirus in the community during winter 2020–21 while strict nonpharmaceutical interventions for COVID-19 were in effect in the city, including work-from-home arrangement for civil servants, universal mask-wearing outside homes, school dismissal, and prohibition on group gatherings of >4 persons in public places (Figure, panel A).

## Conclusions

An abrupt decrease in activities of multiple diarrheagenic viruses, in particular norovirus and rotavirus, and shortening of their seasons was observed soon after the initial global spread of COVID-19 during early 2020. Hong Kong has adopted a multipronged elimination strategy to contain COVID-19 since the first imported case in late January 2020 (*9*) and maintained one of the world’s lowest severe acute respiratory syndrome coronavirus 2 infection rates so far (<0.2% of the local population). If one considers that viral gastroenteritis was primarily transmitted through person-to-person contact, nonpharmaceutical interventions for COVID-19, such as social distancing, might have inadvertently stopped the spread of nonrespiratory pathogens. 

Universal mask-wearing might have also reduced the transmission risk for norovirus, which can reportedly spread by the airborne route (*10*) and vomiting (*11*). The dramatic reduction in virus-positive rates to barely detectable levels in winter 2019–20 is not likely to be an artifact of underreporting because the corresponding number of stool specimens tested was only moderately reduced. Although the return of viral gastroenteritis is anticipated in countries implementing mitigation strategy accompanied with relaxation of infection control measures, the seasonal activities of norovirus and rotavirus observed in winter 2020–21 in Hong Kong were to some extent unexpected because major nonpharmaceutical interventions were still in force during that period, as in winter 2019–20. This finding is unlikely to be explained by pandemic fatigue because local seasonal influenza activity remained at an unprecedented virtually zero level during winter 2020–21 (*12*). Other factors, such as waning immunity and thus accumulation of susceptible population, might come to play.

This study is limited by the lack of virus characterization to determine whether the increase in viral gastroenteritis was a result of emergence of new strains, especially for norovirus, in which new immune-escaped strains emerged periodically (*13*). There were no reports of new and rapidly spreading norovirus variants detected during the COVID-19 pandemic. Additional analysis on the route of transmission of cases would be helpful because public health interventions for COVID-19 might be less effective for diarrheagenic viruses that can spread by foodborne or waterborne routes.

In conclusion, control measures for COVID-19 may have inadvertently reduced the activities of multiple diarrheagenic viruses to barely detectable levels in winter 2019–20. However, norovirus and rotavirus activity returned in winter 2020–21 to levels similar to that in the pre–COVID-19 period. The initial collateral benefit of nonpharmaceutical interventions for COVID-19 that reduced the burden of viral gastroenteritis is not sustainable even in a city with stringent social distancing and continual zero COVID-19 control strategy.
